# Responsiveness of the Arabic Quick Disabilities of the Arm, Shoulder and Hand in Patients with Upper Extremity Musculoskeletal Disorders

**DOI:** 10.3390/healthcare11182507

**Published:** 2023-09-10

**Authors:** Mishal M. Aldaihan, Ali H. Alnahdi

**Affiliations:** Department of Rehabilitation Sciences, College of Applied Medical Sciences, King Saud University, P.O. Box 10219, Riyadh 11433, Saudi Arabia

**Keywords:** psychometrics, upper extremity, outcome assessment, health care, patient reported outcome measures, functional status

## Abstract

This study aimed to examine the responsiveness of the Arabic Disabilities of the Arm, Shoulder and Hand short version (Quick-DASH) in patients with upper extremity musculoskeletal disorders. Participants with upper extremity musculoskeletal disorders (*N* = 88) under physical therapy care were assessed at initial visit and later at a follow-up visit, and they completed the Arabic Quick-DASH, DASH, Numeric Pain Rating Scale (NPRS), Global Assessment of Function (GAF), and the Global Rating of Change Scale (GRC). Responsiveness of the Arabic Quick-DASH was assessed by examining six pre-defined hypotheses. Consistent with the pre-defined hypotheses, the Arabic Quick-DASH changes scores exhibited significant positive correlation with the change in DASH (r = 0.98), GAF (r = 0.67), NPRS (r = 0.72), and the GRC (r = 0.78). As hypothesized, the Arabic Quick-DASH showed a large effect size above the pre-determined level (ES = 1.61, SRM = 1.49) in patients who reported improved upper extremity function. The Arabic Quick-DASH change score discriminated between patients who reported improvement versus no improvement in upper extremity function (area under the receiver operating characteristic curve = 0.90). The results supported 100% (six out of six) of the pre-defined hypotheses. The Arabic Quick-DASH demonstrated sufficient responsiveness where all the pre-defined hypotheses were supported, leading to the established validity of the Arabic Quick-DASH change score as a measure of change in upper extremity function and symptoms. The minimal importance change in the Arabic Quick-DASH needs to be determined in future studies.

## 1. Introduction

Upper extremity musculoskeletal disorders including the shoulder, elbow, or wrist and hand are common disorders [1,2]. Upper extremity musculoskeletal disorders are commonly associated with functional limitations in daily tasks that require the use of the upper extremity [3,4,5]. Assessing the extent of activity limitations and monitoring changes in the magnitude of these limitations from the patients’ perspective during the course of intervention is an important aspect of patient-centered care recommended by the current clinical practice guidelines for patients with upper extremity musculoskeletal disorders [6,7,8,9].

The short version of the Disabilities of the Arm, Shoulder and Hand (Quick-DASH) is a frequently used patient-reported outcome measure (PROM) to assess upper extremity activity limitations and symptoms [10]. The Quick-DASH has been reported to have sufficient structural validity, internal consistency, test–retest reliability, and construct validity [11]. The measurement properties of the Arabic version of the Quick-DASH has been examined in patients with upper extremity musculoskeletal disorders [12]. The Arabic Quick-DASH showed sufficient structural validity, internal consistency, test–retest reliability, and construct validity, but its responsiveness was not examined [12].

The consensus-based standards for the selection of health measurement instruments (COSMIN) define responsiveness as “the ability of a health-related patient-reported outcome instrument to detect change over time in the construct to be measured” [13]. Examining the ability of the Arabic Quick-DASH to detect a change over time in the construct to be measured (upper extremity activity limitation and symptoms) require establishing the validity of the Arabic Quick-DASH change scores as a measure of change in upper extremity activity limitation and symptoms [14,15]. Kennedy et al., in their systematic review, questioned the responsiveness of Quick-DASH and some of its adapted versions and noted that a number of the studies they reviewed lack rigorous methodology in examining Quick-DASH responsiveness [11]. More recent research studies with appropriate methodology reported sufficient responsiveness of Quick-DASH in patients with upper extremity musculoskeletal disorders [16,17,18,19,20].

Given the lack of prior studies examining the responsiveness of the Arabic Quick-DASH, this study aimed to examine the responsiveness of the Arabic Quick-DASH in patients with upper extremity musculoskeletal disorders. We hypothesized that the Arabic Quick-DASH would be a responsive measure in patients with upper extremity musculoskeletal disorders. 

## 2. Materials and Methods

### 2.1. Study Design

Prospective cohort study with two measurement time points.

### 2.2. Setting and Participants

Participants attending three outpatient physical therapy clinics in Riyadh city, Saudi Arabia for a primary complaint of upper extremity musculoskeletal disorder were recruited using convenience sampling. Participants were recruited if they had upper extremity musculoskeletal disorder and were 18 years of age or older. Participants were excluded from participation if they were unable to read and understand Arabic language or had spinal, neurological, cardiovascular or pulmonary disorders that cause functional limitations.

### 2.3. Procedure

Participants with upper extremity musculoskeletal disorders were assessed at two time points. The baseline assessment was completed at the patients’ initial visit in the physical therapy clinic, while the follow-up assessment was completed at least one week from the baseline assessment. Participants in the current study received physical therapy treatments between the two testing sessions and the type and details of the physical therapy interventions were solely determined by the treating therapist. At both testing sessions, participants were asked to complete the Arabic Quick-DASH [12], Numeric Pain Rating Scale [21] Disabilities of the Arm, Shoulder and Hand [22], and Global Assessment of Function [12,23]. The Global Rating of Change Scale [24,25] was also completed by all participants in the follow-up testing session.

### 2.4. Outcome Measures

#### 2.4.1. Disabilities of the Arm, Shoulder and Hand Short Version (Quick-DASH) 

The Quick-DASH is an 11-item PROM that measures upper extremity activity limitation and symptoms [10]. Items were scored from 1: indicating no functional limitation and no symptoms, to 5: indicating functional inability and extreme symptoms. The total score was computed by transforming the mean items score to a scale from 0 to 100, where 0 indicates the best upper extremity function and no symptoms. The Arabic version of the Quick-DASH used in the current study has been reported to be valid and reliable in patients with upper extremity musculoskeletal disorders [12].

#### 2.4.2. Disabilities of the Arm, Shoulder and Hand (DASH)

The DASH is a 30-item PROM that measure upper extremity activity limitation and symptoms [26,27]. The DASH items were scored from 1 to 5, where 1 indicates no functional limitation and no symptoms, and 5 indicates functional inability and extreme symptoms. The DASH total score was computed by transforming the mean items score to a scale from 0 to 100, where 0 indicates the best upper extremity function and no symptoms. Evidence of good measurement properties of the Arabic DASH, including reliability, validity, and responsiveness, was reported previously [22].

#### 2.4.3. Numeric Pain Rating Scale (NPRS)

Participants were asked to rate their pain intensity at the site of upper extremity disorder on a scale from 0: suggesting no pain, to 10: suggesting the worst pain imaginable [28]. The Arabic NPRS was reported to be valid and reliable [12,21].

#### 2.4.4. Global Assessment of Function (GAF)

Participants self-reported their ability of performing activities of daily living from 0 (unable to perform any activity of daily living) to 100 (able to perform all activities of daily living without difficulty) [12,29]. The validity and reliability of the GAF in patients with musculoskeletal disorders was established previously [12,29].

#### 2.4.5. Global Rating of Change Scale (GRC)

At the follow-up testing session, participants were asked to rate their perceived change in upper extremity function compared to the baseline testing session. The scores in the GRC ranged from −5 (a very great deal worse) to 5 (a very great deal better) [12,25,29]. Participants were classified as “improved” if they scored 3 or above in the GRC, and classified as “not improved” if they scored 2 or below.

### 2.5. Statistical Analysis

Hypothesis testing was used to examine the responsiveness of the Arabic Quick-DASH in patients with upper extremity musculoskeletal disorders [14,30]. Six hypotheses were defined a priori to examine the responsiveness of the Arabic Quick-DASH (Table 1). The hypotheses were regarding the expected direction and strength of correlation between the Arabic Quick-DASH change scores and the change scores in the comparator instruments, expected magnitude of change in the Arabic Quick-DASH in patients reporting improved function, and regarding the discriminative ability of the Arabic Quick-DASH change score (Table 1). The responsiveness of the Arabic Quick-DASH was considered sufficient if the results supported at least 75% of the pre-defined hypotheses [31,32]. The computations of the change scores of all outcome measures in the current study (Quick-DASH, DASH, NPRS, GAF) were completed so that positive change scores reflect an improvement in upper extremity function and pain, while negative change scores reflect a worsening in upper extremity function and pain. Pearson’s and Spearman’s correlation coefficients were used to examine the correlation between the Arabic Quick-DASH change scores and the change scores in the comparator instruments (Table 1). The Arabic Quick-DASH effect size was computed as follows: $ES=\frac{Difference\;between\;baseline\;and\;follow-up\;mean\;scores}{Baseline\;standard\;deviation}\;$, while its standardized response mean was computed as follows: $SRM=\frac{Difference\;between\;baseline\;and\;follow-up\;mean\;scores}{Standard\;deviation\;of\;change\;scores}\;$ [33].

The Area under (AUC) the receiver operating characteristic curve was used to examine the ability of the Arabic Quick-DASH change score to discriminate between patients who improved according to their GRC scores and those who did not improve [14]. The Receiver operating characteristic curve was constructed by plotting the false positive rate (1-specificity) in the *x*-axis and the true positive rate (sensitivity) in the *y*-axis for multiple Quick-DASH change score thresholds. An AUC of at least 0.7 was used to indicate sufficient ability of the Arabic Quick-DASH to discriminate between the two groups of patients, and therefore support the responsiveness of the Arabic Quick-DASH [31,32]. Dependent *t*-tests were also used to examine the difference in all outcome measures between the baseline and follow-up assessments. All statistical analyses were conducted using IBM SPSS Statistics 26 (IBM Corp, Armonk, NY, USA).

### 2.6. Sample Size Estimation

For examining the responsiveness of a PROM using hypothesis testing, the minimum required sample size was determined to be 50 participants according to the COSMIN recommendations [15]. Thus, 50 participants were considered the minimum required number of participants in the current study.

## 3. Results

**Figure 1:**
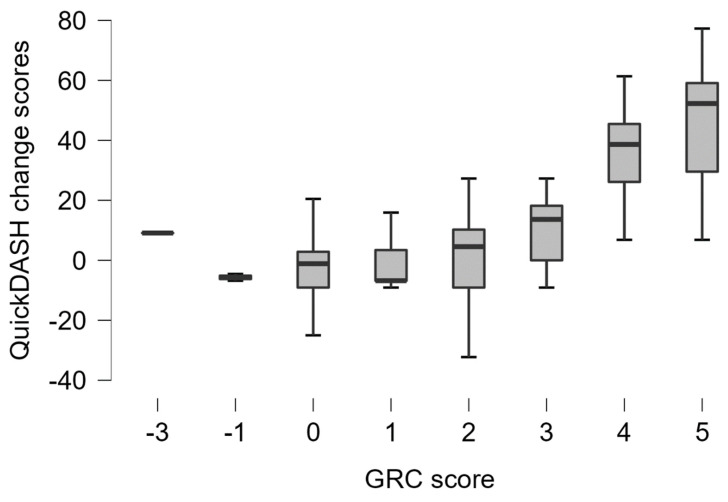
Boxplot showing the Arabic Quick-DASH change scores according to the global rating of change scores.

The current study recruited 88 participants with upper extremity musculoskeletal disorders (Table 2). Baseline, follow-up, and change scores in the Arabic Quick-DASH and other outcome measures are presented in Table 3. At baseline, only one participant had one missing item in the Arabic Quick-DASH (item 10). At follow-up, also one participant had one missing item in the Arabic Quick-DASH (item 3). The Arabic Quick-DASH had no floor or ceiling issues at both baseline and follow-up testing (Table 3). Scores in the Arabic Quick-DASH based on GRC scores are displayed in Figure 1.

All participants completed the baseline and follow-up assessments with a mean time of 19.6 days (Range: 7–72 days) between the two testing sessions. Compared to their baseline assessment, most of the participants (62.5%) reported improvement in upper extremity function (based on GRC scores) at the follow-up assessment, while 36.4% and 1.1% reported no change and a worsening in upper extremity function, respectively (Table 4).

From baseline to follow-up assessments, participants showed a significant reduction (*p* < 0.001) in the Arabic Quick-DASH scores (mean difference: 20.63 points; 95% CI of the difference: 15.22–26.03), a significant reduction (*p* < 0.001) in the DASH scores (mean difference: 20.07 points; 95% CI of the difference: 14.42–25.71), a significant increase (*p* < 0.001) in the GAF scores (mean difference: 20.47 points; 95% CI of the difference: 15.45–25.48), and a significant reduction (*p* < 0.001) in the NPRS scores (mean difference: 2.11 points; 95% CI of the difference: 1.45–2.76) (Table 3).

**Figure 2:**
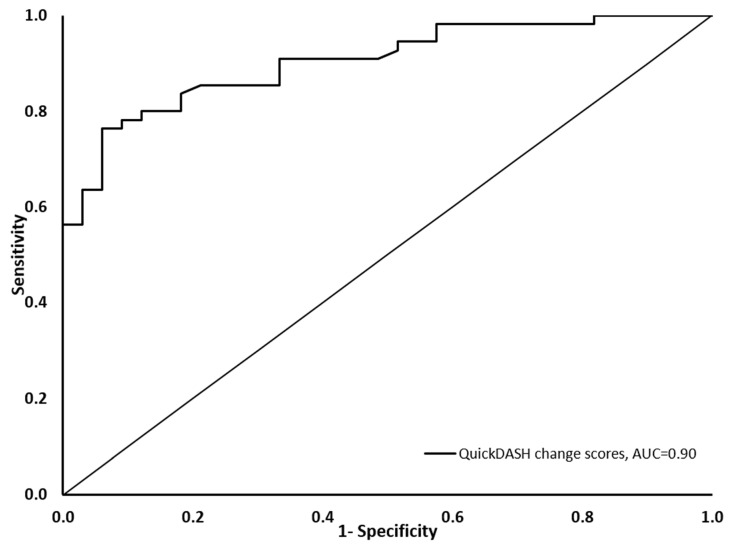
Receiver operating characteristic curve showing the Arabic Quick-DASH sensitivity on the vertical axis and 1-specificity in the horizontal axis.

A significantly positive correlation was observed between the change scores in the Arabic Quick-DASH and the change scores of the NPRS, DASH, and GAF (Table 5). A significantly positive correlation was also observed between the change scores in the Arabic Quick-DASH and the GRC (Table 5). In patients who reported improvement in their upper extremity function (GRC ≥ 3), the Arabic Quick-DASH showed a large effect size above the pre-determined level (Table 3). The Arabic Quick-DASH change score was able to discriminate between patients who reported improvement in their upper extremity function and patients who reported no improvement in their upper extremity function with an AUC of 0.90 (95% CI: 0.84–0.96), which is significantly different (*p* < 0.001) from the null hypothesis of AUC = 0.5, suggesting that discrimination ability is equal to chance (Figure 2).

## 4. Discussion

This study aimed to examine the responsiveness of the Arabic Quick-DASH in patients with upper extremity musculoskeletal disorders. We hypothesized that the Arabic Quick-DASH would be responsive measure in patients with upper extremity musculoskeletal disorders. The results of the current study supported our hypothesis and demonstrated that the Arabic Quick-DASH was a responsive measure of upper extremity function and symptoms.

Six hypotheses were defined prior to data collection to examine the responsiveness of the Arabic Quick-DASH. These hypotheses we defined were based on the argument that change scores in the Arabic Quick-DASH represent change in upper extremity function and symptoms. All of these hypotheses (100%) were supported by the results of the current study, supporting the responsiveness of the Arabic Quick-DASH as a measure of upper extremity function and symptoms.

Quick-DASH is the short version of the DASH, thus correlating the change scores in the Arabic Quick-DASH with that of the DASH was a criterion approach to examine responsiveness where DASH acts as the criterion or gold standard [14,34]. The magnitude of expected correlation between the change scores in the Arabic Quick-DASH and DASH was chosen to be at least 0.7. This threshold is recommended by the COSMIN guideline for criterion validation, and was used in the current study given the criterion approach used in examining responsiveness [31,32]. The results of the current study supported the hypothesized magnitude and direction of the correlation between the Arabic Quick-DASH and the DASH change scores, substantiating the responsiveness of the Arabic Quick-DASH. To the best of our knowledge, this is the first report of the correlation between change in the Quick-DASH and change in the DASH scores with no prior studies having reported such correlation. A number of previous studies have only reported cross-sectional correlations between the Quick-DASH and DASH scores at multiple time points pre and post-interventions, and strong cross-sectional correlations between the two measures [35,36,37,38,39,40].

Pain intensity measured using NPRS in the current study reflects a construct related to the construct measured by the Arabic Quick-DASH, upper extremity function and symptoms. Based on that, it was hypothesized that change scores in both measures would demonstrate, at least, a moderate positive correlation, indicating that reduced pain intensity would be associated with improved upper extremity function. This hypothesized correlation was supported in the current study and reports in previous literature also support this finding. The correlation between change in measures of pain intensity and change in the Quick-DASH, similar to that reported in the current study, was reported in adapted versions of the Quick-DASH, such the Norwegian version (NPRS change, r = 0.62 in patients with shoulder pain) [20], and the Dutch version (Oxford elbow score pain change r = 0.45; SF-36 bodily pain change r = 0.41 in patients with elbow dislocation) [17]. Change scores in other measures of upper extremity function, such as the DASH, upper extremity functional index, and upper extremity functional scale, also exhibited a pattern of correlation with change in measures of pain intensity similar to what is reported between change in the Arabic Quick-DASH and change in NPRS in the current study [27,41].

Currently, in the literature, there is no consistency regarding the number of levels to be used in the GRC, but in the current study, an 11-point GRC was used consistently with the recommended optimal levels in the GRC [25]. In order to be used for validating the change score in the Arabic Quick-DASH as measure of change in upper extremity function, the GRC in the current study was construct specific, asking about change in upper extremity function [14]. The Arabic Quick-DASH change scores and the GRC were hypothesized to have at least moderate positive correlation based on the argument that both reflect change in upper extremity function. This hypothesis was supported by the results of the current study. In line with the hypothesis defined in the current study, previous literature has reported a moderate correlation between change scores in the original English Quick-DASH and GRC (r = 0.45) [42] (r = 0.54, 0.56) [16] in patients with shoulder pain. Additionally, a similar pattern was also reported in the Italian and Norwegian versions of Quick-DASH, where the change scores in these versions showed a correlation with GRC (r = 0.71) [18] (r = 0.47) [20] consistent with the hypothesis and findings of the current study. It is important to note that these comparator studies have used either a 7-point [16,18,20] or 15-point GRC [42], where the current study used an 11-point GRC. In addition, the majority of the comparator studies used GRC that enquire about overall change rather than change in a specific construct [16,18,42], while only one study used a construct-specific GRC, similar to the current study, enquiring about change in shoulder function [20].

The responsiveness of the Arabic Quick-DASH has been supported in the current study by the ability of its change scores to discriminate between patients who reported improvement in their upper extremity function and patients who reported no improvement in their upper extremity function based on GRC scores. The point estimate (AUC = 0.90) and lower limit of this discriminative ability 95% CI (AUC 95% CI: 0.84–0.96) both fall above the recommended threshold (>0.70) to support the PROM responsiveness [31,43]. This discriminative ability suggests that change scores in the Arabic Quick-DASH reflect change in upper extremity function, which is the essence of responsiveness. This ability of the Quick-DASH to discriminate between patients with improved and not improved status was reported previously for the original English Quick-DASH in patients who received physical therapy care for their musculoskeletal shoulder pain (AUC = 0.82) [42] (AUC = 0.78, 0.85) [16]. The discriminative ability of the Arabic Quick-DASH reported in the current study is also consistent with the reported discriminative ability of adapted versions of the Quick-DASH, such the Swedish (AUC = 0.82, in patients who underwent upper extremity surgery) [44], Italian (AUC = 0.86, in patients who received physical therapy care for upper extremity musculoskeletal disorders) [18], Danish (AUC = 0.84, 0.83; in patients who received physical therapy care for shoulder pain) [19], and the Norwegian version (AUC = 0.75; in patients who received physical therapy care for shoulder pain) [20]. Collectively, these prior reports in the literature support the findings of the current study.

ES, and SRM were used in the current study to first determine the magnitude of change that occurred in the Arabic Quick-DASH and the other outcome measures. Based on the magnitude of ES, and SRM, the Arabic Quick-DASH and the other outcome measures demonstrated a large change (>0.8), arguably representing a large improvement in upper extremity function and a large reduction in symptoms [45]. ES and SRM were also used in the current study as part of hypotheses testing, which defined a priori to examine the responsiveness of the Arabic Quick-DASH. This use of the effect size indices as part of hypotheses to be tested is in line with the recommendations of the COSMIN guidelines [14,34]. On the contrary, a number of prior studies have computed the effect size indices and used only the magnitude of these indices to suggest sufficient responsiveness of the Quick-DASH with no pre-specified hypotheses regarding the expected magnitude and direction of the effect size indices [35,36,46]. The magnitude of the effect size indices alone reflects the magnitude of change that occurs and does not reflect the validity of the change scores, which is the responsiveness of the outcome measure [14,34].

In line with the hypothesis and findings of the current study, Quick-DASH was reported to demonstrate a moderate to large effect size for patients under physical therapy care for their upper extremity musculoskeletal disorders, and this was demonstrated in the English Quick-DASH and also in translated versions of Quick-DASH [16,20,47]. In the current study, it was hypothesized that Arabic Quick-DASH in patients with improved upper extremity function (GRC ≥ 3) would demonstrate ES and SRM ≥ 0.5. This magnitude of change was chosen based on the argument that it represents an expected medium improvement in upper extremity function [45]. Results of the current study supported this hypothesized magnitude of change in patients with improved status. Prior reports in literature have reported similar findings, where patients with improved status (according to GRC) demonstrated a large effect size in the Quick-DASH [16,20,47].

Limitations in the current study should be acknowledged. Participants with upper extremity musculoskeletal disorders in the elbow and forearm represent a minority of the sample included, thus caution should be exercised when interpreting findings of the current study for patients with elbow and forearm disorders. The change score representing the minimal importance change in the Arabic Quick-DASH was not determined in the current study. This was not conducted given the known bias in determining the minimal importance change in a sample, like ours, with unequal proportion with improved and not improved patients [48]. Additionally, the sample size in the current study is lower than the recommended sample size for determining a PROM minimal important change [48].

## 5. Conclusions

The Arabic Quick-DASH demonstrated sufficient responsiveness, where all the pre-defined hypotheses were supported leading to established validity of the Arabic Quick-DASH change score as measure of change in upper extremity function and symptoms. Clinicians and researchers are recommended to use the Arabic Quick-DASH to quantify and detect change in upper extremity function and symptoms in patients with upper extremity musculoskeletal disorders.

## Figures and Tables

**Table 1:** Pre-defined hypotheses to examine the Arabic Quick-DASH responsiveness.

Pre-Defined Hypothesis	Hypothesis Supported
1. Positive correlation between the Quick-DASH changes scores and DASH change scores (≥0.7).	Yes
2. Positive correlation between the Quick-DASH changes scores and GAF change scores (≥0.4).	Yes
3. Positive correlation between the Quick-DASH changes scores and NPRS change scores (≥0.4).	Yes
4. Positive correlation between the Quick-DASH changes scores and GRC scores (≥0.4).	Yes
5. Patients reporting improvement (GRC ≥ 3) have Quick-DASH ES and SRM ≥ 0.5	Yes
6. The Quick-DASH discriminates between patients with improved upper extremity function and patients with no improvement in upper extremity function (based on GRC) as indicated by area under the ROC curve (AUC) ≥ 0.70	Yes

**Table 2:** Characteristics of participants (*n* = 88).

Variable	Mean ± SD or *n* (%)
Age (year)	38.47 ± 13.88
Sex	
Male	51 (58.00)
Female	37 (42.00)
Height (m)	1.67 ± 0.09
Mass (kg)	75.97 ± 16.52
Body mass index (kg/m^2^)	27.31 ± 6.04
Site of dysfunction	
Shoulder and arm	36 (40.90)
Elbow and forearm	17 (19.30)
Wrist and hand	35 (39.80)
Upper extremity surgery	
Yes	39 (44.30)
Time after surgery (months)	1.84 (1.84) *
No	49 (55.70)
Duration of symptoms (months)	2.99 (8.75) *

* = median (interquartile range).

**Table 3:** Outcome measures at baseline and follow-up.

Variable	Baseline Scores Mean ± SD	Follow-Up Scores Mean ± SD	Change Scores Mean ± SD	ES	SRM	Baseline		Follow-Up	
						Floor	Ceiling	Floor	Ceiling
Quick-DASH (0–100)	51.57 ± 20.43	30.95 ± 25.2	20.63 ± 25.52	1.01	0.81	0%	0%	0%	14.80%
Improved (*n* = 55)	54.05 ± 20.73	20.63 ± 20.87	33.42 ± 22.41	1.61	1.49				
Unchanged (*n* = 32)	46.65 ± 19.31	47.66 ± 22.90	−1.01 ± 13.25	−0.05	−0.08				
DASH (0–100)	49.98 ± 20.97	29.91 ± 25.11	20.07 ± 26.64	0.96	0.75	0%	0%	0%	13.60%
GAF (0–100)	57.50 ± 18.84	77.97 ± 20.22	20.47 ± 23.67	1.09	0.86	0%	0%	0%	15.90%
NPRS (0–10)	5.06 ± 2.51	2.95 ± 2.86	2.11 ± 3.10	0.84	0.68	4.50%	3.40%	2.30%	30.70%

Quick-DASH = Quick disabilities of the arm, Shoulder and Hand, DASH = Disabilities of the Arm, Shoulder and Hand, GAF = global assessment of function, NPRS = numeric pain rating scale, ES = Effect size SRM = Standardized response mean. Floor represents the percentage of participants with the worst score, representing the worst status, while the ceiling represents the percentage of participants with the best score, representing the best status.

**Table 4:** Participants according to their global rating of change score at follow-up.

Variable	*n* (%)
GRC	
5 (Very great deal better)	25 (28.40)
4 (Great deal better)	15 (17.00)
3 (Moderately better)	15 (17.00)
2 (Little bit better)	15 (17.00)
1 (A tiny bit better, almost the same)	7 (8.00)
0 (No change)	8 (9.10)
−1 (Tiny bit worse, almost the same)	2 (2.30)
−2 (Little bit worse)	0 (0.00)
−3 (Moderately worse)	1 (1.10)
−4 (Great deal worse)	0 (0.00)
−5 (Very great deal worse)	0 (0.00)
Change over time status according to GRC score	
Unchanged vs. Improved vs. Worsened	
Unchanged (GRC −2 to 2)	32 (36.36)
Improved (GRC ≥ 3)	55 (62.50)
Worsened (GRC ≤ −3)	1 (1.14)
Improved vs. Not improved	
Improved (GRC ≥ 3)	55 (62.50)
Not improved (GRC ≤ 2)	33 (37.50)

GRC = global rating of change scale.

**Table 5:** Correlation between the Quick-DASH change score and change in other measures.

Variable	r (95% CI)	*p*
DASH change	0.98 (0.97 to 0.99)	<0.001
GAF change	0.67 (0.49 to 0.80)	<0.001
NPRS change	0.72 (0.61 to 0.81)	<0.001
GRC	0.78 (0.69 to 0.84) *	<0.001

r = Pearson’s correlation coefficient; CI = confidence interval; Quick-DASH = Quick disabilities of the arm, Shoulder and Hand, DASH = Disabilities of the Arm, Shoulder and Hand, GAF = global assessment of function, NPRS = numeric pain rating scale, GRC = global rating of change scale. * = examined using Spearman’s correlation coefficient.

## Data Availability

The data presented in this study are available from the corresponding author on reasonable request.

## Abbreviation

Abbreviation	Full Form
PROM	Patient-Reported Outcome Measure
COSMIN	Consensus-Based Standards for the Selection of Health Measurement Instruments
CI	Confidence Interval
GAF	Global Assessment of Function
GRC	Global Rating of Change Scale
NPRS	Numeric Pain Rating Scale
DASH	Disabilities of the Arm, Shoulder and Hand
ES	Effect Size
SRM	Standardized Response Mean
AUC	Area Under the Curve
SPSS	Statistical Package for the Social Sciences

## References

[1] Huisstede B.M., Bierma-Zeinstra S.M., Koes B.W., Verhaar J.A. (2006). Incidence and prevalence of upper-extremity musculoskeletal disorders. A systematic appraisal of the literature. BMC Musculoskelet. Disord..

[2] Lucas J., van Doorn P., Hegedus E., Lewis J., van der Windt D. (2022). A systematic review of the global prevalence and incidence of shoulder pain. BMC Musculoskelet. Disord..

[3] Vincent J.I., MacDermid J.C., King G.J.W., Grewal R. (2021). The Patient-Rated Elbow Evaluation and the American Shoulder and Elbow Surgeons-Elbow form capture aspects of functioning that are important to patients with elbow injuries. J. Hand Ther..

[4] van Kooij Y.E., Poelstra R., Porsius J.T., Slijper H.P., Warwick D., Selles R.W. (2021). Content validity and responsiveness of the Patient-Specific Functional Scale in patients with Dupuytren’s disease. J. Hand Ther..

[5] Roe Y., Rysstad T., Tveter A.T., Sandbakk T.B., Jaeger M., Grotle M. (2021). What Are the Most Important Problems in Functioning Among Patients with Shoulder Pain? An Analysis of the Patient-Specific Functional Scale. Phys. Ther..

[6] Lucado A.M., Day J.M., Vincent J.I., MacDermid J.C., Fedorczyk J., Grewal R., Martin R.L., Dewitt J., Paulseth S., Dauber J.A. (2022). Lateral Elbow Pain and Muscle Function Impairments. J. Orthop. Sports Phys. Ther..

[7] Erickson M., Lawrence M., Jansen C.W.S., Coker D., Amadio P., Cleary C. (2019). Hand Pain and Sensory Deficits: Carpal Tunnel Syndrome. J. Orthop. Sports Phys. Ther..

[8] Kelley M.J., Shaffer M.A., Kuhn J.E., Michener L.A., Seitz A.L., Uhl T.L., Godges J.J., McClure P.W. (2013). Shoulder pain and mobility deficits: Adhesive capsulitis. J. Orthop. Sports Phys. Ther..

[9] Lin I., Wiles L., Waller R., Goucke R., Nagree Y., Gibberd M., Straker L., Maher C.G., O’sullivan P.P.B. (2020). What does best practice care for musculoskeletal pain look like? Eleven consistent recommendations from high-quality clinical practice guidelines: Systematic review. Br. J. Sports Med..

[10] Beaton D.E., Wright J.G., Katz J.N., Upper Extremity Collaborative Group (2005). Development of the QuickDASH: Comparison of three item-reduction approaches. J. Bone Jt. Surg. Am..

[11] Kennedy C.A., Beaton D.E., Smith P., Van Eerd D., Tang K., Inrig T., Hogg-Johnson S., Linton D., Couban R. (2013). Measurement properties of the QuickDASH (disabilities of the arm, shoulder and hand) outcome measure and cross-cultural adaptations of the QuickDASH: A systematic review. Qual. Life Res..

[12] Alnahdi A.H. (2021). Validity and reliability of the Arabic quick disabilities of the arm, Shoulder and Hand (QuickDASH-Arabic). Musculoskelet. Sci. Pract..

[13] Mokkink L.B., Terwee C.B., Patrick D.L., Alonso J., Stratford P.W., Knol D.L., Bouter L.M., de Vet H.C. (2010). The COSMIN study reached international consensus on taxonomy, terminology, and definitions of measurement properties for health-related patient-reported outcomes. J. Clin. Epidemiol..

[14] de Vet H.C.W., Terwee C.B., Mokkink L.B., Knol D.L. (2011). Measurement in Medicine: A Practical Guide.

[15] Terwee C.B., Mokkink L.B., Knol D.L., Ostelo R.W., Bouter L.M., de Vet H.C. (2012). Rating the methodological quality in systematic reviews of studies on measurement properties: A scoring system for the COSMIN checklist. Qual. Life Res..

[16] Chester R., Jerosch-Herold C., Lewis J., Shepstone L. (2017). The SPADI and QuickDASH Are Similarly Responsive in Patients Undergoing Physical Therapy for Shoulder Pain. J. Orthop. Sports Phys. Ther..

[17] Iordens G.I.T., Hartog D.D., Tuinebreijer W.E., Eygendaal D., Schep N.W.L., Verhofstad M.H.J., Van Lieshout E.M.M., on behalf of FuncSiE Trial Investigators (2017). Minimal important change and other measurement properties of the Oxford Elbow Score and the Quick Disabilities of the Arm, Shoulder, and Hand in patients with a simple elbow dislocation; validation study alongside the multicenter FuncSiE trial. PLoS ONE.

[18] Franchignoni F., Vercelli S., Giordano A., Sartorio F., Bravini E., Ferriero G. (2014). Minimal clinically important difference of the disabilities of the arm, shoulder and hand outcome measure (DASH) and its shortened version (QuickDASH). J. Orthop. Sports Phys. Ther..

[19] Budtz C.R., Andersen J.H., de Vos Andersen N.B., Christiansen D.H. (2018). Responsiveness and minimal important change for the quick-DASH in patients with shoulder disorders. Health Qual. Life Outcomes.

[20] Rysstad T., Grotle M., Klokk L.P., Tveter A.T. (2020). Responsiveness and minimal important change of the QuickDASH and PSFS when used among patients with shoulder pain. BMC Musculoskelet. Disord..

[21] Alghadir A.H., Anwer S., Iqbal Z.A. (2016). The psychometric properties of an Arabic numeric pain rating scale for measuring osteoarthritis knee pain. Disabil. Rehabil..

[22] Alotaibi N.M., Aljadi S.H., Alrowayeh H.N. (2016). Reliability, validity and responsiveness of the Arabic version of the Disability of Arm, Shoulder and Hand (DASH-Arabic). Disabil. Rehabil..

[23] Alnahdi A.H., Alrashid G.I., Alkhaldi H.A., Aldali A.Z. (2016). Cross-cultural adaptation, validity and reliability of the Arabic version of the Lower Extremity Functional Scale. Disabil. Rehabil..

[24] Aljathlani M.F., Alshammari M.O., Alsuwaygh M.A., Al-Mutairi M.S., Aljassir F.F., Bindawas S.M., Alnahdi A.H. (2022). Cross-cultural adaptation and validation of the Arabic version of the upper extremity functional index. Disabil. Rehabil..

[25] Kamper S.J., Maher C.G., Mackay G. (2009). Global rating of change scales: A review of strengths and weaknesses and considerations for design. J. Man. Manip. Ther..

[26] Hudak P.L., Amadio P.C., Bombardier C. (1996). Development of an upper extremity outcome measure: The DASH (disabilities of the arm, shoulder and hand) [corrected]. The Upper Extremity Collaborative Group (UECG). Am. J. Ind. Med..

[27] Beaton D.E., Katz J.N., Fossel A.H., Wright J.G., Tarasuk V., Bombardier C. (2001). Measuring the whole or the parts? Validity, reliability, and responsiveness of the Disabilities of the Arm, Shoulder and Hand outcome measure in different regions of the upper extremity. J. Hand Ther..

[28] Hawker G.A., Mian S., Kendzerska T., French M. (2011). Measures of adult pain: Visual Analog Scale for Pain (VAS Pain), Numeric Rating Scale for Pain (NRS Pain), McGill Pain Questionnaire (MPQ), Short-Form McGill Pain Questionnaire (SF-MPQ), Chronic Pain Grade Scale (CPGS), Short Form-36 Bodily Pain Scale (SF-36 BPS), and Measure of Intermittent and Constant Osteoarthritis Pain (ICOAP). Arthritis Care Res..

[29] Alnahdi A.H. (2021). Measurement properties of the 15-item Arabic lower extremity functional scale. Disabil. Rehabil..

[30] Mokkink L.B., de Vet H.C.W., Prinsen C.A.C., Patrick D.L., Alonso J., Bouter L.M., Terwee C.B. (2018). COSMIN Risk of Bias checklist for systematic reviews of Patient-Reported Outcome Measures. Qual. Life Res..

[31] Prinsen C.A.C., Mokkink L.B., Bouter L.M., Alonso J., Patrick D.L., de Vet H.C.W., Terwee C.B. (2018). COSMIN guideline for systematic reviews of patient-reported outcome measures. Qual. Life Res..

[32] Prinsen C.A., Vohra S., Rose M.R., Boers M., Tugwell P., Clarke M., Williamson P.R., Terwee C.B. (2016). How to select outcome measurement instruments for outcomes included in a “Core Outcome Set”—A practical guideline. Trials.

[33] Portney L.G., Watkins M.P. (2009). Foundations of Clinical Research: Applications to Practice.

[34] Mokkink L.B., Terwee C.B., Knol D.L., Stratford P.W., Alonso J., Patrick D.L., Bouter L.M., de Vet H.C. (2010). The COSMIN checklist for evaluating the methodological quality of studies on measurement properties: A clarification of its content. BMC Med. Res. Methodol..

[35] Rodrigues J., Zhang W., Scammell B., Russell P., Chakrabarti I., Fullilove S., Davidson D., Davis T. (2016). Validity of the Disabilities of the Arm, Shoulder and Hand patient-reported outcome measure (DASH) and the Quickdash when used in Dupuytren’s disease. J. Hand Surg. Eur. Vol..

[36] Macdermid J.C., Khadilkar L., Birmingham T.B., Athwal G.S. (2015). Validity of the QuickDASH in patients with shoulder-related disorders undergoing surgery. J. Orthop. Sports Phys. Ther..

[37] Cao S., Zhou R., Zhou H., Chen Y., Cui H., Lu Z., Qian Q., Ding Y. (2019). Reliability and validity of Simplified Chinese version of Quick Disabilities of the Arm, Shoulder, and Hand (QuickDASH) questionnaire: Cross-cultural adaptation and validation. Clin. Rheumatol..

[38] da Silva N.C., Chaves T.C., dos Santos J.B., Sugano R.M.M., Barbosa R.I., Marcolino A.M., Mazzer N., Fonseca M.C.R. (2020). Reliability, validity and responsiveness of Brazilian version of QuickDASH. Musculoskelet. Sci. Pract..

[39] Kc S., Sharma S., Ginn K., Reed D. (2021). Measurement properties of the Nepali version of the Quick-DASH in patients with shoulder pain. Musculoskelet. Sci. Pract..

[40] Aasheim T., Finsen V. (2014). The DASH and the QuickDASH instruments. Normative values in the general population in Norway. J. Hand Surg. Eur. Vol..

[41] Chesworth B.M., Hamilton C.B., Walton D.M., Benoit M., Blake T.A., Bredy H., Burns C., Chan L., Frey E., Gillies G. (2014). Reliability and validity of two versions of the upper extremity functional index. Physiother. Can..

[42] Mintken P.E., Glynn P., Cleland J.A. (2009). Psychometric properties of the shortened disabilities of the Arm, Shoulder, and Hand Questionnaire (QuickDASH) and Numeric Pain Rating Scale in patients with shoulder pain. J. Shoulder Elb. Surg..

[43] Terwee C.B., Bot S.D.M., de Boer M.R., van der Windt D.A.W.M., Knol D.L., Dekker J., Bouter L.M., de Vet H.C.W. (2007). Quality criteria were proposed for measurement properties of health status questionnaires. J. Clin. Epidemiol..

[44] Gummesson C., Ward M.M., Atroshi I. (2006). The shortened disabilities of the arm, shoulder and hand questionnaire (QuickDASH): Validity and reliability based on responses within the full-length DASH. BMC Musculoskelet. Disord..

[45] Cohen J. (2013). Statistical Power Analysis for the Behavioral Sciences.

[46] Dale L.M., Strain-Riggs S.R. (2013). Comparing responsiveness of the quick disabilities of the arm, shoulder, and hand and the upper limb functional index. Work.

[47] Polson K., Reid D., McNair P.J., Larmer P. (2010). Responsiveness, minimal importance difference and minimal detectable change scores of the shortened disability arm shoulder hand (QuickDASH) questionnaire. Man. Ther..

[48] Terwee C.B., Peipert J.D., Chapman R., Lai J.-S., Terluin B., Cella D., Griffith P., Mokkink L.B. (2021). Minimal important change (MIC): A conceptual clarification and systematic review of MIC estimates of PROMIS measures. Qual. Life Res..

